# Seroprevalence and associated factors of brucellosis and Q-fever in cattle from Ibarapa area, Oyo State, South-western Nigeria

**DOI:** 10.11604/pamj.2020.36.370.24925

**Published:** 2020-08-29

**Authors:** Simeon Idowu Cadmus, Kelechi Araloluwa Akporube, Fiyinfoluwa Ola-Daniel, Olubukola Deborah Adelakun, Victor Oluwatoyin Akinseye

**Affiliations:** 1 Department of Veterinary Public Health and Preventive Medicine, Faculty of Veterinary Medicine, University of Ibadan, Ibadan, Oyo State, Nigeria,; 2 Center for Control and Prevention of Zoonoses, Faculty of Veterinary Medicine, University of Ibadan, Ibadan, Oyo State, Nigeria,; 3 Department of Veterinary Public Health and Preventive Medicine, College of Veterinary Medicine, Michael Okpara University of Agriculture, Umudike, Nigeria,; 4 Department of Animal Health Technology, College of Agriculture and Technology, Igboora, Oyo State, Nigeria,; 5 Department of Chemical Sciences, Augustine University Ilara-Epe, Lagos State, Nigeria

**Keywords:** Brucellosis, Q-fever, seroprevalence, Ibarapa area, Oyo State

## Abstract

**Introduction:**

a cross-sectional study was conducted to determine the seroprevalence and associated factors of brucellosis and Q-fever among cattle in a rural setting in Oyo State, Nigeria.

**Methods:**

one hundred and fourty nine serum samples (24 males; 125 female) from 16 cattle herds were collected and screened. The Rose Bengal Plate test (RBPT) and competitive Enzyme-Linked Immunosorbent Assay (cELISA) were used for brucellosis while indirect Enzyme-Linked Immunosorbent Assay (iELISA) was used for Q-fever. Further, a checklist was used to collect data on cattle sampled. Data were analyzed using STATA 12.

**Results:**

serum analysis revealed that 11.4% (17/149) and 6.7% (10/149) were seropositive by RBPT and cELISA respectively for brucellosis, while 23.5% (35/149) were seropositive by iELISA for Q-fever. A significant association was detected between cattle age (OR=27.7; 95% CI: 2.34-449.86), herd size (OR=10.53; 95% CI: 1.85-60.53) and seropositivity to Brucella infection. Also, there was a significant association between breed (OR=6.69; 95% CI: 1.7-28.74), herd size (OR=4.25; 95% CI: 1.31-13.85) of cattle and seropositivity to Coxiella burnetii infection. Importantly, the only significant associated factor to cattle herd seropositivity to Brucella and C. burnetii infections was the method of handling aborted foetuses.

**Conclusion:**

the study revealed that brucellosis and Q-fever are prevalent among cattle in the study area. Thus, there is a need for further studies to provide better insight into the epidemiology of both diseases and particularly Q-fever. This becomes imperative in the study area and generally in Nigeria given the dearth of information about the diseases in pastoralist communities who are at grave risk of infection at the human-animal-ecosystem interface.

## Introduction

Brucellosis and Q-fever (coxiellosis) are two important zoonotic diseases of livestock responsible for a variety of clinical conditions leading to infertility and significant economic losses [1]. Brucellosis is a disease of major public health importance as it is highly contagious and frequently caused by the consumption of unpasteurized milk/milk products, ingestion of meat from infected animals, or through contact with either infected animals or their secretions [2-4]. Brucellosis is caused by bacteria of the genus *Brucella*; and B. melitensis is considered to be the most virulent in humans, followed by *B. abortus* and *B. suis* [5]. The disease is widely distributed in Africa with the highest incidence in areas where extensive livestock husbandry is practised coupled with a high animal population [6]. Notably, uncontrolled free movement of animals practised by the nomadic Fulani herdsmen has been associated with the spread of the disease [7]. In livestock, it is primarily a reproductive disease characterized by late abortion, retained foetal membranes, orchitis and impaired fertility [8]. Other diseases, however, can also cause abortion such as Q-fever which remains under-reported in Africa [4]. Q-fever is also known as coxiellosis and is a highly contagious zoonotic disease caused by the intracellular pathogen *Coxiella burnetii*. It is known for its high persistence, infectivity and has a worldwide distribution except for New Zealand [9]. Animals may become infected by direct contact with infected animals and contaminated environments or from the inhalation of aerosolized bacteria [4]. Infected animals can contaminate the environment by shedding organisms through milk, faeces, urine, saliva, vaginal secretions and other products of conception [10]. Transmission of this infection to humans is mainly through the inhalation of contaminated aerosols, but can also occur after consumption of infected unpasteurised milk and dairy products [11]. In Nigeria, brucellosis has been reported extensively in ruminants [3,4,12,13] whereas only limited studies exist on *C. burnetii* infections in ruminants in the country [4,14,15]. Remarkably, co-infection of both brucellosis and Q-fever can occur in both humans and animals with resultant negative outcomes. The aim of this study, therefore, was to determine the seroprevalence of brucellosis and Q-fever and the associated factors influencing the presence of *Brucella spp*. and *C. burnetii* antibodies in cattle in Ibarapa area of Oyo State, south-western Nigeria.

## Methods

**Study design:** a cross-sectional study was carried out to detect infections with *Brucella spp*. and *C. burnetii* and the associated factors that influence their presence among pastoralist cattle herds. The study was carried out between June and August 2018 in Eruwa and Igbo-Ora in Ibarapa, Oyo State, Nigeria.

**Figure 1:**
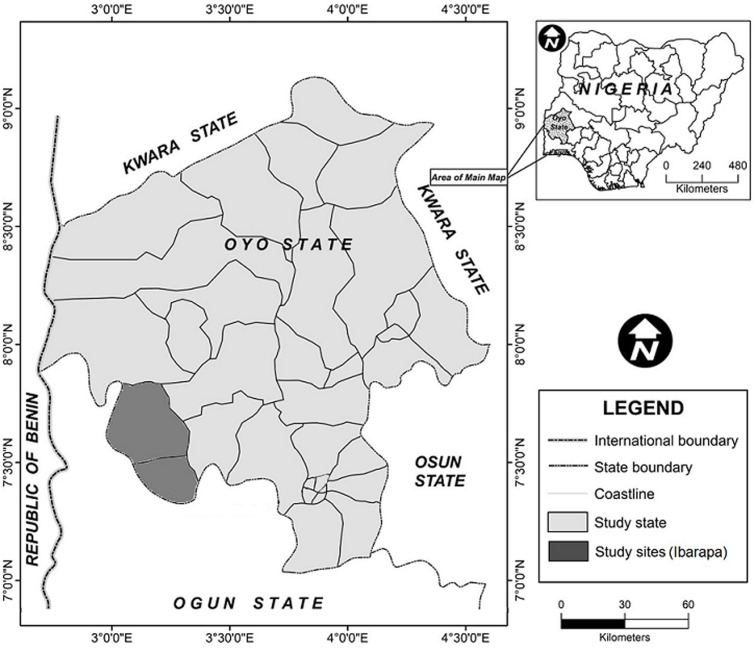
map showing the locations in Oyo State where herds where sampled

**Study area:** this study was conducted in Ibarapa area of Oyo State (Figure 1). The Ibarapa area falls within latitudes 70.15´N and 70.55´N and longitudes 30´E and 30.3´E. It is located approximately 100km North of the coast of Lagos and about 95km west of Ibadan, Oyo State capital. Eruwa and Igbo-Ora the two towns where the study was carried out are part of “Ibarapa-Meje”, a term popularly used to describe the congregation of seven towns within three local government areas (LGAs) in Ibarapa, Oyo State. Many pastoralist settlements and their cattle herds are situated in these towns because of the availability of green pastures all year round

**Sampling and sample size determination:** multistage random sampling was used to select the cattle herds screened. The LGAs of Ibarapa North, East and Central formed the first stratum, the towns in each LGA formed the second stratum, the pastoralist settlements the third stratum and the herds in each settlement the fourth stratum. In each stratum, the simple random sampling technique was used. Sampling within each herd was also done using simple random sampling, taking into consideration the age and sex of the animals. Of the three LGAs of Ibarapa, two were selected (Ibarapa East and Central) from which one town each (Eruwa and Igbo-Ora) were selected and a total of 16 cattle herds sampled from both towns. The required number of animals to be sampled was calculated using an expected prevalence (Pexp) of 10.1% [13] for brucellosis and 6.2% [4] for Q-fever, a confidence interval (CI) of 95% and a desired absolute precision of 5%. The sample size was calculated using:

$$n=\frac{Z^{2}P\text{exp(1-}P\text{exp)}}{d^{2}}$$

The calculated sample size for brucellosis was 140 samples while that of Q-fever was 90 samples. Since each sample obtained was analyzed for both diseases, the higher sample size obtained for brucellosis was used. Therefore, the overall sample size was 140 samples.

**Sample collection and handling:** blood collection was carried out by veterinarians who are part of the study team. Each sample was properly labelled with relevant information such as the age, sex, breed and body condition recorded using unique codes. A checklist was further used to gain information on the herd size and management practices from the owner of each herd sampled. The blood samples were transported in ice packs to the tuberculosis and brucellosis research laboratories of the Department of Veterinary Public Health and Preventive Medicine, University of Ibadan. Here, the samples were later centrifuged, the serum harvested and stored at -20°C until further laboratory analysis was carried out.

**Serological tests:** the serological tests conducted were the Rose Bengal Plate test (RBPT) and competitive Enzyme-Linked Immunosorbent Assay (cELISA) for brucellosis; while the indirect Enzyme-linked Immunosorbent Assay (iELISA) was used for Q-fever. The RBPT was carried out as earlier described [16] while the cELISA and iELISA were performed according to the manufacturer´s instructions. The antigens for the RBPT and the cELISA kits for brucellosis were sourced from the Animal and Plant Health Agency (APHA), United Kingdom, while the iELISA ID screen® Q-fever indirect multispecies test kits were sourced from innovative diagnostics (ID vet) Montpellier, France.

**Statistical analysis:** data were analysed using STATA version 12. Descriptive statistics were carried out and group differences were tested using chi-square statistics for categorical variables. The main outcome measure was a positive animal (RBPT for brucellosis and iELISA for Q-fever). A multivariable unconditional logistic regression was carried out using all variables that were statistically significant at 30% level in the bivariate analysis. All tests were two-tailed and statistical significance was set at p≤0.05.

**Ethical statement:** the ethical approval for this study was obtained from the Animal Care and Use Research Ethics Committee, University of Ibadan, Nigeria (Ref: UI-ACUREC 18/0078).

## Results

**Demographic characteristics of the sampled cattle:** of the 149 cattle screened, most 125 (83.9%) were female while majority 127 (85.2%) were two years and above. Most 131 (87.9%) of the cattle were Bunaji breed and a large percentage (82.4%) of the cattle had good body condition while the majority (45.6%) of the herd screened were in the category of 31-50 heads of cattle (Table 1). The distribution of cattle screened according to individual animal prevalence showed that out of the 149 sera tested, 11.4% (17/149) and 6.7% (10/149) were seropositive to *Brucella* antibodies by RBPT and cELISA, respectively; 23.4% (35/149) were seropositive to Q-fever and 6.04% (9/149) were seropositive to co-infection with both diseases. Out of the 16 cattle herds screened, 31% (5/16) were seropositive to *Brucella* antibodies, 50% (8/16) were seropositive to Q-fever and 31% (5/16) were seropositive to co-infection with both diseases.

**Table 1:** characteristics of the cattle screened from pastoralist settlements (N=149)

Characteristics	Frequency (N)	Percentage (%)
**Sex**		
Male	24	16.1
Female	125	83.9
**Age**		
Adult (>2years)	127	85.2
Young adult (6months-2years)	22	14.8
**Breed**		
Bunaji	131	87.9
Sokoto Gudali	8	5.4
Cross breed	10	6.7
**Herd Size**		
10-30	42	28.2
31-50	68	45.6
50 above	39	26.2

**Seroprevalence and factors associated with *Brucella* infection:** bivariate analysis for RBPT showed that cow (OR=3.37, 95% CI: 1.08-10.57) and cattle (OR=3.02, 95% CI: 1.12-9.18) older than two years of age have higher odds of being seropositive to brucellosis. Crossbreed cattle, those with poor body condition and those in the >50 herd size show higher likelihood of being seropositive to brucellosis (OR=4.67, 95% CI: 2.28-9.54), (OR=5.14, 95% CI: 2.34-9.45) and (OR=8.89, 95% CI: 3.23-24.51) (Table 2). The multivariable logistic regression revealed that age of cattle and herd size are important associated factors for *Brucella* infection in cattle screened. Cattle older than two years of age are more likely to be seropositive to *Brucella* antibodies compared to those younger than two years (OR=27.7, 95% CI: 2.34-44.9). Cattle in herd size of >50 show a higher likelihood of being seropositive to brucellosis when compared to those within 10-30 herd size (OR=10.53, 95% CI: 1.85-60.53) (Table 3). At the herd level, the only significant associated risk factor was the method of handling aborted foetuses; the herds that bury and/or burn aborted foetuses are less likely (OR=0.03, 95% CI: 0.001-0.65) to be seropositive compared to those that throw them away in the bush (Table 4).

**Table 2:** seroprevalence of brucellosis (Brucella spp) and Q fever (Coxiella burnetii) in individual cattle screened according to sex, age, breed and herd size

	Brucellosis				Q fever infection			
Characteristics	Positive (n)%	OR	95% CI	P-value	Positive (n)%	OR	95% CI	P-value
**Sex**								
Male	1(4.17)	1			7(29.17)	1		
Female	16(12.80)	3.37	1.08-0.57	0.05^*^	28(22.4)	0.7	0.37-1.33	0.35
**Age**								
>2 years	16(12.6)	3.02	1.12- 9.18	0.05^*^	30(23.62)	1.05	0.55-2.03	0.90
0-2years	1(4.55)	1			5(22.73)	1		
**Breed**								
Bunaji	12(9.16)	0.71	0.29-1.73	0.59	24(18.32)	1		
Sokoto Gudali	1(12.50)	1			5(62.50)	7.43	3.89-4.21	0.00^*^
Cross breed	4(40.00)	4.67	2.28-9.54	0.00^*^	6(60)	6.69	3.51-2.75	0.00^*^
**Body condition**								
Poor	3(17.65)	5.14	2.34-9.45	0.00^*^	18(51.43)	4.67	0.67-8.56	0.18
Good	14(82.35)	1			17(48.57	1		
**Herd Size**								
10-30	2(4.76)	1			4(9.52)	1		
31-50	3(4.41)	0.92	0.25-3.48	0.8	23(33.82)	4.86	2.21-0.66	0.00^*^
50 above	12(30.77)	8.89	3.23-4.51	0.00^*^	8(20.51)	2.45	1.07-5.60	0.04^*^

Statistical significance was set at p ≤ 0.05; No: number, OR: odds ratio, ^*^statistically significant

**Table 3:** results of logistic regression analysis of variables associated with individual animal seropositivity to *Brucella spp* and *Coxiella burnetii* antibodies

	Brucellosis			Q fever infection		
Variable	OR	95%CI	P-value	OR	95%CI	P-value
**Breed**						
Bunaji	1			1		
Sokoto Gudali	13.60	0.80-22.8	0.07	5.93	1.20-27.95	0.02^*^
Cross breed	4.96	0.67-36.21	0.11	6.99	1.71-28.74	0.00^*^
**Age**						
<2 years	1					
≥2 years	27.7	2.34-44.9	0.01^*^	1.8	0.49-6.75	0.36
**Herd Size**						
10-30	1			1		
31-50	0.73	0.09-5.46	0.75	4.25	1.31-13.85	0.02^*^
50 above	10.53	1.85-60.53	0.01^*^	1.90	0.49-7.41	0.35

Statistical significance was set at p≤0.05. No: number, OR: odds ratio, ^*^statistically significant

**Table 4:** results of logistic regression analysis of significant variables associated with cattle herd seropositivity to *Brucella spp* and *Coxiella burnetii* antibodies

	Brucellosis			Q fever infection		
Variable	OR	95%CI	P-value	OR	95%CI	P-value
**Herd size**						
0-50	1			1		
>50	3.07	0.07-12.79	0.55	1.7	0.07-4.27	0.73
**Method of handling of aborted foetuses**						
Throw away in the bush	1			1		
Bury and/or burn	0.03	0.001-0.65	0.02^*^	0.1	0.03-0.83	0.04^*^

Statistical significance was set at p≤0.05; No: number, OR: odds ratio, ^*^statistically significant

**Seroprevalence of *Coxiella burnetii*:** for Q-fever, the bivariate analysis showed an association between seropositivity, breed of cattle and herd size. Sokoto Gudali and crossbreed cattle show a higher likelihood of being seropositive to *C. burnetii* antibodies (OR=7.43, 95% CI: 3.89-14.21); (OR=6.69, 95% CI: 3.51-12.75). Furthermore, cattle within herds that are 31-50 (OR=4.86, 95% CI: 2.21-10.6) and >50 in size (OR=2.45, 95% CI: 1.07-5.60) show higher likelihood of seropositivity to *C. burnetii* antibodies. The multivariable logistic regression revealed that the breed of cattle and herd size are important associated factors for the occurrence of Q-fever in cattle screened. Importantly, Sokoto Gudali and crossbreed cattle are 13.6 and almost five times more likely to be infected with *C. burnetii* (OR=5.93, 95% CI: 1.20-27.95); (OR=6.99, 95% CI: 1.71-36.21) when compared to Bunaji breed. Again, those cattle within-herd size of between 31-50 are more likely to be infected when compared with herds of 10-30 in size (OR=4.25, 95% CI: 1.31-13.85). Notably, at the herd level, the only identified associated factor was the method of handling aborted fetuses. Thus, cattle within herds that bury and/or burn aborted foetuses are less likely (OR=0.1, 95% CI: 0.03-0.83) to be infected with *C. burnetii* compared to those that throw them away in the bush (Table 4).

## Discussion

**Seroprevalence of *Brucella* infection:** a seroprevalence of 11.4% and 6.7% was obtained in this study through RBPT and cELISA respectively. The seroprevalence recorded could be as a result of the insufficient official policies put in place for the control of brucellosis in Nigeria [17], which is complicated by the unrestricted movement of livestock within the study area and from neighbouring African countries [18]. Furthermore, this could be linked to lack of awareness on the mode of transmission of the disease among the herdsmen [19], which in turn leads to infected animals with signs of the disease being retained within the herds [3]. The brucellosis seroprevalence was however lower than the reports of 18.5% in Kaduna State, Nigeria [4] and 12.91% in Kenya [20] but higher than reports of 10.1% in some other settings in Southwestern Nigeria [13]. The nomadic system practised among the Fulani herdsmen in Nigeria has been identified as a major risk factor for *Brucella* infection [3]. This study indicate that brucellosis is prevalent among cattle screened in the study area. This is of grave public health concern because of the close association between cattle and humans and the general habits of consuming unpasteurised milk and milk products in the Ibarapa area. The prevalence reported with RBPT (11.4%) was higher than that of cELISA (6.7%). This is in agreement with the findings of other studies [3,12,21]. This is possible because serological tests differ in their ability to detect a particular immunoglobulin [22].

IgM and IgG1 are the isotypes of the immunoglobulin found in the blood in early or acute infections [23] and this may not be seen in chronic, recurrent and relapse cases [24]. The RBPT is said to be better suited for the detection of acute cases [25] (i.e. the IgM and IgG1) while the cELISA is best suited for chronic infections [26]. Importantly, the RBPT has also been reported to be superior to the cELISA [27,28]. Thus, it is recommended as the test of choice in endemic and resource-poor countries where vaccination is not generally practised like Nigeria because of its simplicity, relatively low cost and high standard in testing for brucellosis [29]. Adult (>2 years) cattle had higher seroprevalence (12.6%) to brucellosis and this was statistically significant. This result agrees with the study earlier carried out in Kaduna State, Northcentral Nigeria [4]. A plausible reason for this is the prolonged exposure of animals to *Brucella spp*. in an infected herd [13]. Notably, brucellosis can occur in all the age groups of cattle but is commonly known to persist in sexually mature animals [30]. Further, age is seen as a factor related to the host that influences the animal´s susceptibility to infection by *Brucella* [31]. The highest seroprevalence was observed in herds with animals >50 (30.77%) than those with smaller sized herds. This is consistent with other findings in Nigeria [3,13,32]. This could be due to an increase in exposure to *Brucella spp*. as a result of a movement from one location to another and interaction with other infected herds at grazing and watering points [3,32].

It has also been shown that large-sized herds have increased exposure to *Brucella* infections following calving and abortion by animals infected with *Brucella* within the herds as a result of higher stocking density in comparison to small-sized herds [27]. The logistic regression analysis revealed that age and herd size of cattle were significant factors associated with individual animal seropositivity to *Brucella* antibodies. Animals older than two years (i.e. adults) are more likely (OR=27.7; 95% CI: 2.34-449.86) to be infected with *Brucella spp*. compared to the young adults. Furthermore, large herd size of more than 50 cattle are more likely (OR=10.53; 95% CI: 1.85-60.53) to be infected with *Brucella* compared to those with fewer than 30 cattle in a herd. Notably, the factor that was significantly associated with cattle herds being seropositive to *Brucella* antibodies in the study was the method of handling aborted foetuses (OR=0.03; 95% CI: 0.001-0.65). Significantly, herds where aborted foetuses were buried and burnt, were less likely to be seropositive to *Brucella* antibodies than those that threw the aborted foetuses into the bush. Further, improper disposal of infected aborted materials was related to the transmission and persistence of brucellosis within cattle herds and among animals. Importantly, the infectivity of the organism can be maintained in the environment for long periods in favourable medium, sunlight, pH and temperature [13].

**Seroprevalence of *C. burnetii*:** seroprevalence of 23.5% was obtained for *C. burnetii* in this study. A seroprevalence higher than the 14.5% reported in Kaduna State, Nigeria [15], 10.5% in Western Kenya [33] and 6.2% reported in another study in Kaduna State, Nigeria [4] but lower than what was obtained (28.3%) in another study in Western Kenya [34]. The prevalence of Q-fever is usually underestimated [35] in sub-Saharan Africa, where data on it are very few. However, it is a common zoonotic disease in most parts of the world with variations in epidemiological data from one country to another. Our findings show that the seroprevalence was higher in the Sokoto Gudali breed (62.5%) than the other breeds. This is in contrast with the findings of earlier studies carried out in Kaduna State, Nigeria [4,15]. The moderately sized herds showed higher seroprevalence (33.82%) when compared to those with small herds and herds with 50 cattle and above. The earlier finding has shown that the bigger the herd size, the higher the risk of transmission of the disease as a result of contact with other infected animals or herds during grazing on infected pasture or the sharing of watering points [4]. Importantly, the logistic regression analysis identified breed and herd size as significant factors associated with individual animal seropositivity to infection of cattle with *C. burnetti*. Further, Sokoto Gudali (OR= 5.93; 95% CI: 1.7-28.74) and the crossbreeds (OR=6.69; 95% CI: 1.7-28.74) of cattle are more likely to be seropositive compared to the Bunaji breed. Also, herds with sizes 31-50 (OR=4.25; 95% CI: 1.31-13.85) showed higher odds than either those with a herd size of 10-30 or those 50 and above. The only significant associated factor with cattle herds being seropositive to *C. burnetii* antibodies in the study was the method of handling aborted foetuses (OR=0.1; 95% CI: 0.03-0.83).

Herds where aborted foetuses were buried and burnt, were less likely to be seropositive to *C. burnetii* antibodies than those that threw the aborted foetuses into the bush as seen for brucellosis. Overall, this study showed a high seroprevalence of Q-fever among the animals screened. Reasons for this could be due to the general lack of awareness of Q-fever among the pastoralists, the large numbers of animals in each herd, the sharing of water and feeding spaces and the open grazing system practised by the pastoralists. Again, the method of disposal of the aborted foetuses could cause further spread of the disease. This study reiterates the assertion that cattle are reservoirs of *C. burnetii* and thus pose serious public health implications for the zoonotic transmission of the disease to humans particularly in Nigeria where the awareness about the disease is poor. Importantly, this study also reveals a seroprevalence of 6% co-infection of both brucellosis and Q-fever among the animals screened. A plausible reason for this may be adduced to the cases of abortion and retained placenta recorded in all the herds screened based on the feedback of the interview conducted among the animal owners using the checklist. Consequently, the simultaneous presence of these infections in cattle herds implies concurrent exposure to both zoonotic diseases that are of grave public health importance. Overall, some limitations were encountered during the fieldwork and these included the language barrier among herd owners as well as the uncooperative attitudes of some of the herd owners. These limitations hindered the screening of more animals in each settlement visited.

## Conclusion

The present study confirmed the presence of *Brucella* and *C. burnetii* antibodies in cattle screened in Eruwa and Igbo-Ora communities in Ibarapa, Oyo State. With the seroprevalence of 11.4% and 6.7% for brucellosis and 23.5% for Q-fever, this raises serious public health concern. This becomes even more important given the similar routes of transmission of both diseases. This situation is further exacerbated by the likelihood of infection of both pastoralists and the general public through the consumption of unpasteurized milk and milk products and the overall lack of awareness about Q-fever. Importantly, based on the inherent public health risks that brucellosis and Q-fever pose, we advocate more enlightenment programmes targeted at the pastoralists on the zoonotic transmission of both diseases and the need to pasteurize milk and milk products. Finally, there should be a better-integrated government and multisectoral approach towards the prevention and control of both zoonotic diseases in Nigeria and other endemic countries of the world.

### What is known about this topic

- Brucellosis and Q-fever are both primarily livestock diseases;
- Brucellosis and Q-fever are both zoonotic and have significant public health importance;
- Brucellosis and Q-fever are diseases with global distribution and are responsible for abortion in livestock.

### What this study adds

- The presence of Brucella and C. burnetii antibodies in pastoralist cattle was confirmed in Ibarapa, Oyo State, Nigeria;
- The study suggests that cattle above two years of age and herds with more than 50 cattle have more likelihood to be seropositive to Brucella antibodies;
- Study revealed that the breed of cattle and herd size are important associated factors for the occurrence of Q-fever in cattle screened.
